# Developing a COVID-19 Mortality Prediction (CoMPred) Indicator for ICU Diabetic Patients Treated with Tocilizumab in Saudi Arabia: A Proof-of-Concept Study

**DOI:** 10.3390/biomedicines11102649

**Published:** 2023-09-27

**Authors:** Anwar A. Sayed, Omar M. Al Nozha

**Affiliations:** 1 Department of Medical Microbiology and Immunology, College of Medicine, Taibah University, Madina 42353, Saudi Arabia; 2 Department of Medicine, Taibah University, Madina 42353, Saudi Arabia; 3 Department of Medicine, Saudi German Hospital, Madina 42373, Saudi Arabia

**Keywords:** CoMPred, COVID-19, death, diabetes mellitus, ICU, mortality prediction, NLR, Saudi Arabia

## Abstract

Since the beginning of the COVID-19 pandemic, efforts have been made to underline its discourse and identify factors contributing to its severe forms. Clinically, many physicians depended on subjective criteria to determine its severe forms, which varied significantly between practices. However, they did not rely on objective laboratory findings. This study aimed to present a novel and objective laboratory-based indicator to predict mortality among COVID-19 patients. The study included 249 COVID-19 patients who were admitted to the ICU, of which 80 did not survive. The COVID-19 Mortality Prediction (CoMPred) indicator was developed by including the age and the following lab investigations: neutrophil-to-lymphocyte ratio (NLR), D-Dimer, PT, aPTT, ESR, CRP, and urea levels. A CoMPred score of 7.5 or higher carries a sensitivity of 81.10% in predicting mortality, i.e., a patient with a CoMPred score of 7.5 or higher has an 81.10% chance of dying. The CoMPred indicator score directly correlates with mortality, i.e., the higher the score, the higher the possibility of the patient dying. In conclusion, the CoMPred indicator is an objective tool that is affordable and widely available, will assist physicians, and limit the burden on clinical decisions on an unpredicted course of COVID-19 in patients.

## 1. Introduction

Since the novel corona virus disease 2019 (COVID-19) took the world by storm, extensive efforts and measures have been taken to limit its impact. Published modelling studies have shown that combining quarantine with protective measures like wearing face masks and implementing travel restrictions was highly effective in reducing the emergence of new COVID-19 cases, transmission of the virus between people, and mortalities secondary to COVID-19 infection [1,2]. Saudi Arabia was one of the earliest countries to impose strict and progressive measures to tackle the COVID-19 pandemic, including social distancing, face masks, and travel restrictions, in addition to a massive vaccination programme, which demonstrated to be successful [3]. COVID-19 mortality rates in Saudi were lower than many countries in both case fatality, which was as low as 1.2%, and in deaths in the entire population (both confirmed cases and healthy population), with one of the lowest rates at 27.6 deaths per 100,000, compared to the US and Canada, for example, which had a mortality rate of 341.11 and 135.23 per 100,000, respectively [4].

Although the mass vaccination programmes significantly alleviated most of the COVID-19 danger, it did not succeed in eliminating the infection entirely, and the mortality rates were still higher than other countries in Saudi [5].

As a viral infection, treating COVID-19 was mostly supportive, with no antiviral treatments that were found to be effective in eliminating it [6]. Moreover, the infection discourse of COVID-19 cases is unpredictable and highly variable between patients [7].

Many clinicians have attempted to determine indicators of disease severity, which included patients’ age, the presence of comorbidity, and other clinical features [8]. Studies have also attempted to evaluate the value of imaging modalities, e.g., chest X-ray and CT scans, in determining disease severity [9,10]. However, a consensus is still to be made around these parameters and every country adapts its own protocol in categorizing the severity of COVID-19 cases.

Scientists have attempted to study the biological parameters during COVID-19 infections in order to extrapolate which of them are indicators of severity, and subsequently, mortality. These parameters included simple and widely available investigations, such as neutrophil-to-lymphocyte ratio (NLR) and platelet count obtained from a complete blood count (CBC) [11,12,13]. More advanced and less available parameters were also studied, e.g., serum interleukins, anti-S protein antibody titers and next-generation sequencing [14]. Although many markers were found to be useful in determining COVID-19 severity, and inferentially, mortality, these are still lacking assessment in implementation in clinical practice.

In this study, we aim to a develop a COVID-19 mortality prediction (CoMPred) score based on laboratory investigations that are widely available and were previously reported to be of value in determining disease severity.

## 2. Materials and Methods

### 2.1. Study Setting

This study included 249 patients who were diagnosed with COVID-19 based on a positive nasopharyngeal quantitative polymerase chain reaction (qPCR) test. Patients were admitted to a tertiary hospital between March 2020 and September 2022, according to the Saudi Ministry of Health (MoH) Hospital admission criteria for COVID-19 patients version 1.1 [15]. Briefly, any symptomatic patients with one of the following criteria were to be admitted: clinical evidence of pneumonia; age older than 65 years; low oxygen saturation on pulsometer less than 94% on room air; acute respiratory distress syndrome; chronic pulmonary or kidney disease; and history of comorbidities or morbid obesity (BMI equal or more than 40). Those without a confirmed COVID-19 qPCR test were not included in the study.

All included patients were admitted to the intensive care unit (ICU) and managed according to the Saudi MoH Protocol for Patients Suspected of/Confirmed with COVID-19 [15]. Those patients showed clinical signs of pneumonia, e.g., fever, cough, dyspnoea, and one of the following: respiratory rate more than 30/min, oxygen saturation on pulsometer less than 93% on room air, or severe respiratory distress.

No personal or sensitive patients’ information were collected as part of this study. This study was conducted in accordance with the declaration of Helsinki and ethically approved from the Taibah University College of Medicine Research Ethics Committee no. TU-21-010.

### 2.2. The Development of the COVID-19 Mortality Prediction (CoMPred) Scoring

The elements included in the CoMPred scoring were chosen based on their reported significance in determining the severity of COVID-19 cases in previously published studies. These includes patients’ age, D-Dimer, prothrombin time (PT), activated partial thromboplastin time (aPTT), NLR, erythrocyte sedimentation rate (ESR), C-reactive protein (CRP), and plasma urea levels. Each element was given a score of 1 if its value exceeded a threshold; otherwise, it would receive 0, except for patients’ age and NLR. The scoring of patients’ age was 0 if they were younger than 50 years old, 1 if the patient was 50 years or older, and 2 if they were 65 years or older. The scoring of the NLR is based on 2 cut-offs: 4 (which would receive a score of 1) and 8 (which would receive a score of 2). These 2 cut-offs for NLR were based on previous studies showing the association of higher NLR with severe forms of disease [11,12]. The breakdown of the CoMPred scoring is demonstrated in Table 1.

### 2.3. Statistical Analysis

Absolute numbers and percentages were used to describe categorical data, e.g., gender and nationality. Descriptive statistics were used to describe numerical data, such as age and laboratory findings, according to their data distribution. The Shapiro–Wilk test was used to determine the data distribution, i.e., whether it follows a normal gaussian distribution (parametric) not (nonparametric). Means and standard deviations were used to describe parametric data, whereas medians and interquartile ranges were used for nonparametric data.

Paired tests were used to compare between the laboratory findings at baseline and at 10-day follow-up. Once the elements of the CoMPred indicator were chosen, a receiver operating characteristic (ROC) curve was made and the area under the curve was calculated to determine the indicator’s sensitivity and specificity. A *p* value of less than 0.05 was considered statistically significant.

## 3. Results

### 3.1. Patients’ Characteristics

Two hundred and forty-nine ICU patients were included in this study. Their average age was 59.87 (±16.88) years-old, with a majority of 70.28% being male patients (*n* = 175). A total of 61.45% of the patients were Saudi nationals (*n* = 153), while the remaining patients were of various nationalities, including Egyptians (*n* = 25), Bangladeshis (*n* = 14), and Pakistanis (*n* = 10).

Patients’ median duration of stay at the ICU was 17 days, of which 67.87% of the patients (*n* = 169) survived in the ICU. A. total of 67.47% of patients were diabetics (*n* = 168), and the majority (94.37%, *n* = 235) received Tocilizumab in addition to the standard Ministry of Health treatment protocol [15]. The breakdown of patients’ characteristics is presented in Table 2.

### 3.2. The Biological Changes in COVID-19 Patients Admitted to the ICU

A baseline lab investigation was conducted of all patients and was carried out daily or more often if needed to monitor the progression of their condition. Laboratory findings indicating recovery included a significant increase in platelet count and a reduction in inflammatory markers, such as CRP and ESR and coagulation profile, e.g., activated partial thromboplastin time (aPTT).

On the other hand, laboratory findings indicating disease progression included a rise in the NLR, urea, and D-dimer levels. The detailed breakdown of patients’ laboratory findings is described in Table 3.

For the purpose of the study, we used the baseline investigations carried out on the day of admission and the follow-up investigations performed 10 days later.

### 3.3. Identifying the Elements Needed for the Development of the CoMPred Indicator

The main outcome that is used in this study to stratify patients is their survival from the infection. Hence, the patients were divided into survivor and non-survivor groups based on their outcome. Then, patients’ baseline investigations were compared between the survivor and non-survivor groups to determine which parameters would be most useful in the CoMPred indicator, as shown in Table 4, as well as based on their reference values.

**Figure 1:**
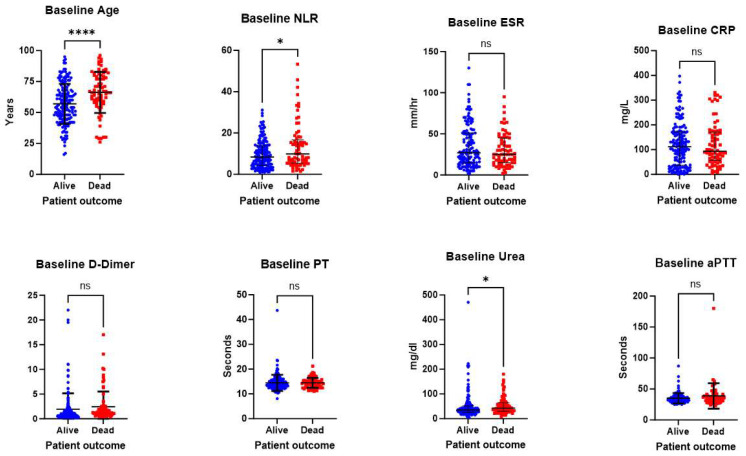
Comparison of patients’ laboratory parameters at baseline. Figures compare the various parameters between survivors (blue) and non-survivors (red). The horizontal bars (black) represent the median and interquartile range. * *p* value < 0.05, **** *p* value < 0.0001, ns: not significant.

Patients’ age and several laboratory parameters (at baseline) were included in CoMPred, which were NLR, CRP, ESR, PT, aPTT, and urea level as shown in Figure 1.

**Figure 2:**
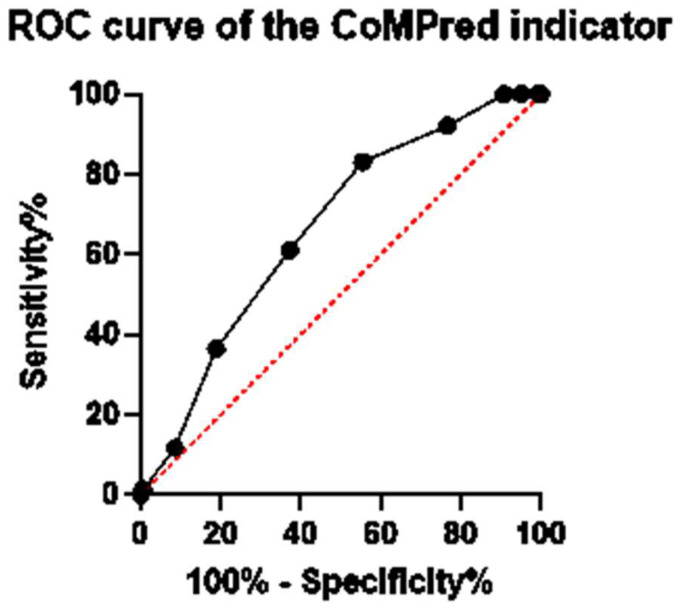
ROC curve of the CoMPred indicator.

Attempts were made to include all possible patients’ characteristics and laboratory investigations that could increase the sensitivity of the CoMPred indicator. These include the patients’ gender, diabetic status, and all the investigations described in Table 3. Interestingly, the inclusion of these parameters reduced the area under the ROC curve, indicating a less accurate CoMPred indicator. Based on our findings described in Figure 1 and previously reported studies, the markers that were chosen to be included in the CoMPred indicator are as follows: patients’ age, NLR, ESR, CRP, Urea, PT, aPTT, and D-dimer. Based on these factors, the CoMPred indicator had an area under the curve of 0.66 at a significance level of less than 0.0001, indicating its capability of distinguishing between survivors and non-survivors, as shown in Figure 2.

A CoMPred score of 7.5, and higher, carries a sensitivity of 81.10% in predicting mortality. From a clinical point of practice, a patient with a CoMPred score of 7.5 or higher has an 81.10% chance of dying. The CoMPred indicator score is directly correlated with mortality, i.e., the higher the score, the higher the possibility of the patient dying. For example, a patient with a CoMPred score of 9 has an over 90% possibility of dying.

## 4. Discussion

Since the beginning of the COVID-19 pandemic, healthcare systems have strived to develop prognostic tools or markers that could predict which group of patients are those who would progress rapidly or have a higher risk of mortality. Many treatment protocols have been tried throughout the pandemic with conflicting results, and even for the protocols with proven benefits, they continue to be without a definitive treatment and still rely mainly on supportive care. While COVID-19 was initially believed to be a solitary respiratory ailment, growing evidence has indicated its systemic nature, suggesting it extends beyond mere respiratory disorder. Hence, depending on a single affected system or laboratory investigation is not favourable, and a comprehensive approach should be adopted to reach best predictive models.

The mortality rate (32.13%) in our studied population was a bit lower than the average international mortality rate (35.5%) and far lower than many other Middle East-reported ICU mortality rates (55.5–74%) [16], which could contribute to the early introduction of Tocilizumab treatment in our protocol that has been reported to reduce mortality rates in critically ill COVID-19 patients and especially those who were ventilated [17].

Age is an independent risk factor for the severity of COVID-19. This stems from the biological changes leading to the gradual deterioration of the immune system, also known as immunosenescence [18]. These changes include low repertoire, shorter telomere length, and structural changes within immune organs. Given the inverse correlation between age and the fitness of the immune response, the age score in the developed indicator is further classified into over 45 and 65 based on the current literature [19].

As a systematic inflammatory condition, it was expected that haematological indices would be influenced and to be used in the CoMPred indicator. Such indices include NLR, ESR, and CRP. NLR has been extensively studied in the context of COVID-19. Our previous multicentre study demonstrated the prognostic value of NLR in detecting severe COVID-19 cases, i.e., admitted to the ICU, compared to both non-severe cases and non-COVID-19 patients [11]. Several studies confirmed such findings both locally [20,21] and internationally [12,22]. In more recent studies, Regolo and colleagues demonstrated the value of NLR as an independent predictor of mortality in separate studies including over 1000 patients with COVID-19 [23,24]. A proper immune response requires an intricate interplay between innate and adaptive immune systems, and hence, the high levels of NLR may reflect a sign of deranged interplay between these systems [25]. In this study, NLR had the capacity to predict mortality on its own, but it also contributed to the increased AUC of the CoMPred indicator. NLR remains an invaluable tool, both on its own and as part of the CoMPred indicator, given its wide availability and cost effectiveness in comparison to other laboratory investigations [26].

The renal involvement in COVID-19 is a sign of severe infection and systemic inflammation, usually referred to as multisystem inflammatory syndrome [27], which could be used to predict mortality. The findings in our study of increased ESR, CRP and Urea are all in lines with the work of teams led by Zeng [28], Ibnouf [29], Abo-Haded [30], and Yarijani [31]. Although both ESR and CRP are inflammatory markers that could increase in other infectious diseases, e.g., TB [32], their inclusion in the CoMPred indicator increased its sensitivity to predicting mortality among COVID-19 patients.

One of the major complications of COVID-19 infection is its unexpected effect on the coagulation profile, leading to a series of thrombotic events in almost 10% of COVID-19 patients [33]. Such complications are sufficiently dangerous, requiring anticoagulation therapy [34].The abnormally high coagulation markers, such as D-dimer and PT, could indicate the development of disseminated intravascular coagulation (DIC) as a result of COVID-19 infection. Interestingly, coagulation markers in our study did not predict mortality on their own, despite being the most common coagulation marker of severity in COVID-19 [35]. However, their inclusion in the CoMPred indicator further increased its area under the ROC curve, hence improving its ability to predict mortality.

Early studies have considered diabetes mellitus as an independent risk factor for COVID-19 severity and mortality [36,37]. This may be attributed to the lack of immunological control of the infection, leading to an uncontrolled hyperinflammatory state of disease [38]. While this may be the case, the inclusion of diabetes in the CoMPred indicator lessened its ability to predict mortality. This could be due to the study population, all being ICU patients, which were already considered to have severe COVID-19. Hence, diabetes could differentiate between severe and non-severe but not predict mortality. Furthermore, hyperglycaemia is the main contributing factor in worsening the prognosis of COVID-19 among diabetic patients [39]. Hence, the treatment of our study cohort in the ICU, ensuring a tight glycaemic control, could have negated the effect of diabetes on the discourse of COVID-19 infection.

The participants of this study were COVID-19 patients who were admitted to the ICU over a period of two years. In these two years, several variants of the virus were reported, such as Delta and Omicron [40]. The evolution and appearance of such variants may alter the pathophysiology and course of the disease [41]. In our cohort, the patients were only confirmed to be infected with COVID-19, but its variant was not determined. It is a possibility that our cohort represents a heterogeneous population of patients with variable pathophysiology [42,43]. The appearance of novel variants of COVID-19 in the near future may change the pathophysiology of the condition. Subsequently, the sensitivity of the CoMPred indicator may change accordingly, either toward an increase or a decrease.

This study introduces a new tool, the CoMPred indicator, which is necessary especially for conditions like COVID-19, a condition that is notoriously of unexpected discourse. Many studies have described several biological changes that occur as part of the COVID-19 pathophysiology, such as T and B cell phenotypes [44,45,46,47], genetic signatures [48], and epigenetics mechanisms [49]. Although these changes could be used for prognostic purposes, they are expensive, with very limited availability in clinical facilities, especially in low or middle-income countries and in remote areas. Subsequently, such unavailability limits their utility and application in clinical practice. The CoMPred indicator provides a cheaper alternative, whose components are affordable and widely available. Furthermore, the indicator provides a comprehensive and objective prognostic tool, alternative to the subject clinical judgement of a physician, which may vary significantly between practitioners of the same clinical establishment.

Despite the strengths of the study and the new insights it provides, the study is not without limitations. Firstly, the study is a retrospective cross-sectional study, which is at risk of sampling bias [50]. Another noted limitation of the study is its nature of being a single-site study. The inclusion of a single site may introduce selection bias; however, it ensures that all patients have received the same management, eliminating the level of care variability and excluding it from being an influencing factor. Another limitation of the study is the lack of acute respiratory distress measures, such as the ratio of arterial oxygen partial pressure (PaO2) to fractional inspired oxygen (FiO2), referred to as the P/F ratio, and patients’ history of vaccination, which may have altered their disease progression. Finally, the tool was developed based on findings from patients admitted to ICU, i.e., developed from severe patients, which may not apply to patients with less severe forms of COVID-19.

Future studies should aim to further build on the findings of this study and address the limitations of this study. For example, a prospective multicentre study should be conducted to assess the sensitivity and specificity of the CoMPred indicator. Future studies should also compare the CoMPred indicator to other well-established scoring systems, such as the Sequential Organ Failure Assessment (SOFA) [51], the Acute Physiology and Chronic Health Evaluation (APACHE II) [52], or the Charlson Comorbidity Index [53].

## 5. Conclusions

In conclusion, the present study presents a novel tool, the CoMPred indicator, which will assist physicians in providing care for patients with COVID-19. The tool is affordable, widely available in clinical settings, and should limit the burden on clinical decisions regarding an unpredicted condition such as COVID-19. Future studies should attempt to validate the usability and value of the CoMPred tool in predicting severe cases and mortality and possibly compare it to other indicators, such as the SOFA and APACHE II.

## Figures and Tables

**Table 1:** Elements of the CoMPred Scoring system.

Element (Unit)	Cut-off Level	Assigned Score
Patient’s age (years)	<50	0
	50–64	1
	≥65	2
D-Dimer (mg/L)	<0.5	0
	≥0.5	1
PT (seconds)	<13.5	0
	≥13.5	1
aPTT (seconds)	<35	0
	≥35	1
NLR	<4	0
	≥4 < 8	1
	≥8	2
ESR (mm/hr)	<30	0
	≥30	1
CRP (mg/L)	<10	0
	≥10	1
Urea (mg/dL)	<35	0
	≥35	1

aPTT: activated partial thromboplastin time; CRP: C-reactive protein; ESR: erythrocyte sedimentation rate; NLR: neutrophil-to-lymphocyte ration; PT: Prothrombin time.

**Table 2:** Patients’ characteristics.

Characteristics (Unit)	Values
Age (years)	59.87 (±16.88) ^
Gender	Male: 175
	Female: 74
Nationality	Saudi: 153 (61.45%)
	Non-Saudi: 96 (38.55%)
ICU admission Outcome	Alive: 169 (67.87%)
	Dead: 80 (32.13%)
Use of Tocilizumab	Yes: 235 (94.37%)
	No: 14 (5.63%)
Diabetic	Yes: 168 (67.47%)
	No: 81 (32.53%)
Duration of stay (days)	17 (11–23.75) ^^

^ Mean (± standard deviation); ^^ Median (interquartile range).

**Table 3:** Patients’ laboratory investigations at baseline and follow-up.

Characteristics	Baseline	Follow-up	*p* Value
WBC (×10^3^/mL)	8.44 (5.8–12.01)	10.32 (7.32–14.83)	<0.0001 ****
RBC count	4.86 (4.38–5.26)	4.74 (4.14–5.34)	0.2
Hemoglobin	13.32 (11.96–14.6)	13.07 (11.42)	0.25
Hematocrit	41 (37.05–44.25)	39.80 (34.60–43.35)	0.2
MCV	85.40 (81–88.95)	84.90 (81.20–88.95)	0.65
MCH	28 (26.10–29.60)	27.90 (26.10–29.50)	0.95
MCHC	32.80 (31.65–33.90)	32.90 (31.75–34)	0.94
RDW	13.20 (12.30–14.38)	13.60 (12.60–14.50)	0.0002 ***
Platelet count	236 (178–314.2)	292.1 (188–387.4)	<0.0001 ****
Mean platelet volume	8.6 (7.18–9.6)	8.6 (7.35–9.5)	0.92
Neutrophil count	7.62 (4.42–11)	8.57 (5.74–12.80)	0.0006 ***
Lymphocyte count	0.77 (0.56–1.22)	0.76 (0.54–1.3)	0.65
NLR	8.86 (4.89–15.37)	11.87 (5.85–19.28)	0.0077 **
Monocyte count	0.35 (0.23–0.55)	0.48 (0.29–0.72)	<0.0001 ****
Eosinophil count	0.03 (0–0.06)	0.04 (0.01–0.08)	0.0003 ***
Basophil count	0.04 (0.02–0.06)	0.04 (0.02–0.07)	0.004 **
ESR	26 (14–49)	20 (11–39)	<0.0001 ****
CRP	103 (49.81–170.6)	8.02 (3.39–33.83)	<0.0001 ****
Urea	36.38 (27.82–54.63)	55.64 (38.52–96.30)	<0.0001 ****
Creatinine	0.9 (0.77–1.26)	0.85 (0.73–1.3)	0.0039 **
ALT	35 (23–53)	54 (34–91.50)	<0.0001 ****
AST	43 (29–64.50)	42 (28–69.50)	0.65
D-Dimer	1.025 (0.61–1.78)	1.55 (0.9–4)	<0.0001 ****
PT	14 (12.75–15.5)	13.8 (12.3–15.65)	0.92
aPTT	35.1 (30.03–40.88)	31.15 (27.18–38.83)	0.0055 **
INR	1.13 (1.04–1.24)	1.12 (1.04–1.25)	0.56

ALT: alanine transaminase; aPTT: activated partial thromboplastin; AST: aspartate transaminase; CRP: C-reactive protein; ESR: erythrocyte sedimentation rate; INR: international normalised ratio; MCH: mean corpuscular haemoglobin; MCHC: mean corpuscular haemoglobin concentration; MCV: mean corpuscular volume; NLR: neutrophil-to-lymphocyte ratio; RBC: red blood cell; RDW: red cell distribution width; WBC: white blood cell. *p* value is less than ** 0.01, *** 0.001, **** 0.0001.

**Table 4:** Comparison of characteristics and remaining lab investigations between survivors and non-survivors.

Characteristics (Unit)	Survivors (*n* = 169)	Non-Survivors (*n* = 80)	*p* Value
Gender	Male: 124	Male: 54	0.37
	Female: 45	Female: 26	
Nationality	Saudi: 103	Saudi: 48	0.89
	Non-Saudi: 66	Non-Saudi: 32	
Diabetic	Yes: 108	Yes: 61	0.08
	No: 58	No: 19	
Use of Tocilizumab	Yes: 157	Yes: 74	0.99
	No: 12	No: 6	
WBC (×10^3^/mL)	8.70 (5.42–12.10)	8.33 (5.97–11.75)	0.82
RBC count	4.87 (4.38–5.31)	4.7 (4.25–5.10)	0.16
Hemoglobin	13.60 (11.95–14.60)	12.90 (11.85–14.46)	0.15
Hematocrit	41.30 (37.03–44.50)	40.40 (36.95–43.55)	0.36
MCV	85.05 (81.03–88.50)	86.40 (80.95–89.75)	0.13
MCH	27.95 (26.30–29.40)	28 (25.75–29.90)	0.84
MCHC	32.90 (31.80–33.90)	32.50 (31.30–33.90)	0.19
RDW	13.10 (12.20–14)	13.70 (12.75–15)	0.003 *
Platelet count	247.70 (188.50–331.90)	217.60 (148–227.50)	0.009 *
Mean Platelet volume	8.50 (7.14–9.40)	8.90 (7.42–9.70)	0.11
Neutrophil count	7.41 (4.08–10.50)	6.94 (5.09–10.58)	0.83
Lymphocyte count	0.83 (0.59–1.28)	0.71 (0.45–1.14)	0.01 *
Monocyte count	0.40 (0.24–0.60)	0.30 (0.18–0.46)	0.002 *
Eosinophil count	0.03 (0.00–0.07)	0.02 (0.00–0.06)	0.22
Basophil count	0.04 (0.02–0.06)	0.03 (0.02–0.04)	0.63
Creatinine	0.86 (0.76–1.22)	1.03 (0.82–1.4)	0.03 *
ALT	34 (20.50–53)	38 (24–56)	0.39
AST	40 (27.50–56.50)	53 (36–79)	0.0008 *
INR	1.12 (1.04–1.23)	1.15 (1.06–1.27)	0.49

ALT: alanine transaminase; AST: aspartate transaminase; INR: international normalised ratio; MCH: mean corpuscular haemoglobin; MCHC: mean corpuscular haemoglobin concentration; MCV: mean corpuscular volume; RBC: red blood cell; RDW: red cell distribution width; WBC: white blood cell. * Indicates statistically significant difference.

## Data Availability

According to the regulatory policies, raw data is available from the corresponding author upon reasonable request.
